# Development and Validation of a Rapid UPLC-MS/MS Determination of Cereulide in ARA Oil and ARA Powder

**DOI:** 10.3390/toxins18080320

**Published:** 2026-07-23

**Authors:** Xia Cui, Jing Zhang, Zixiao Zhou, Ziyi Wang, Jing Hou, Rong Zhao, Sai Fan

**Affiliations:** 1 Beijing Key Laboratory of Diagnostic and Traceability Technologies for Food Poisoning, Beijing Center for Disease Prevention and Control, Beijing 100013, China; cuixiastyle@163.com (X.C.);; 2 Qinghai Center for Disease Prevention and Control, Xining 810021, China

**Keywords:** cereulide, ARA oil, ARA powder, HLB-P, UPLC-MS/MS

## Abstract

Arachidonic acid (ARA) is an essential long-chain polyunsaturated fatty acid for infant growth and development, commonly added to infant formula and supplementary foods as ARA oil or ARA powder. Since December 2025, infant formula products have been recalled in several countries due to cereulide contamination in ARA ingredients. Cereulide, a heat-resistant emetic toxin produced by *Bacillus cereus*, presents a serious threat to infant health. However, specific analytical methods for cereulide detection in ARA raw materials remain scarce, hindering effective quality control and source prevention of food safety hazards. This study developed and validated a rapid ultra-performance liquid chromatography–tandem mass spectrometry (UPLC-MS/MS) method for the quantitative determination of cereulide in ARA oil and powder. Sample pretreatment was optimized according to matrix characteristics: for microencapsulated ARA powder, water dissolution was performed to release cereulide before acetonitrile extraction, whereas direct extraction was applied to ARA oil. Subsequent HLB-P pass-through solid-phase extraction effectively eliminated matrix interference in both matrices. The method exhibited excellent linearity (*R*^2^ > 0.999) from 0.1 to 20 μg·L^−1^, with an LOD of 0.03 μg·kg^−1^ and an LOQ of 0.1 μg·kg^−1^, respectively. Spike recoveries reached 90.7–107.0% for ARA oil (RSDs ≤ 6.5%) and 88.5–107.9% for ARA powder (RSDs ≤ 7.0%). This rapid and reliable method provides a critical analytical tool to monitor cereulide in ARA raw materials and mitigate infant safety hazards at the source.

## 1. Introduction

Foodborne toxins pose a sustained and serious threat to global public health [1,2,3]. Cereulide, a heat-stable cyclic dodecapeptide toxin produced by certain *Bacillus cereus* strains, is a well-recognized causative agent of acute foodborne emetic poisoning in humans [4,5]. Upon ingestion, it elicits typical gastrointestinal symptoms, including nausea, severe vomiting, and abdominal discomfort. In severe cases, intoxication can advance to multiple organ failure and even threaten patient survival [4,6,7,8]. Beyond its acute toxicity, chronic low-dose cereulide exposure can activate endoplasmic reticulum (ER) stress, triggering intestinal inflammation, gut microbiota dysbiosis, and impaired serotonin synthesis [9]. Moreover, cereulide may exert synergistic toxic effects with other co-occurring toxins, further exacerbating intestinal tissue injury [10]. Cereulide exhibits extraordinary thermal stability and maintains bioactivity even after routine food processing and disinfection operations, including pasteurization, making conventional thermal sterilization ineffective. Due to its remarkable thermal stability and high contamination risk, cereulide contamination in food and raw materials poses a persistent and intractable challenge to global food safety management [11].

Since December 2025, large-scale recalls of infant formula products have been initiated across multiple countries and regions worldwide, with all incidents attributed to cereulide contamination, which has aroused extensive public concerns and regulatory attention. Subsequent official traceability investigations confirmed that arachidonic acid (ARA) raw materials were the key suspected contaminant source [12]. As an essential long-chain polyunsaturated fatty acid supporting infant neurological and retinal development [13], ARA is widely incorporated into infant formulas and complementary foods in the form of oil or microencapsulated ARA powder [14]. In response to the emerging cereulide contamination risk in ARA-related raw materials for infant formula, the European Union (EU) has revised its safety regulatory standards. The European Commission (EC) set an acute reference dose (ARfD) of 0.014 μg·kg^−1^ body weight for cereulide in infant formula [15] and officially issued Regulation (EU) 2026/459 on 24 February 2026, requiring that all ARA products imported into the EU be accompanied by an official certificate verifying the non-detection of cereulide, with formal enforcement starting from 28 February 2026.

The new regulatory requirements urgently demand reliable detection technologies for cereulide in ARA raw materials. However, matrix-specific detection approaches tailored to ARA oil and ARA powder remain scarce in current academic research, representing a critical technical gap in food safety surveillance. Liquid chromatography–tandem mass spectrometry (LC-MS/MS) is widely acknowledged as the gold-standard technique for trace cereulide detection due to its superior accuracy, sensitivity, specificity, and quantitative performance and has been successfully applied to cereulide analysis in various food matrices, including rice and dairy products [16,17,18,19]. Nevertheless, distinct from conventional complex food matrices, the unique physicochemical properties of ARA oil and ARA powder pose specific challenges that existing LC-MS/MS methods fail to address, necessitating the development of matrix-specific methodologies.

Compared with conventional complex food matrices, including infant formula, ARA oil and ARA powder have relatively simple chemical compositions. However, they possess unique and distinct physicochemical properties that bring unique challenges to sample pretreatment. As a pure lipid-based matrix, ARA oil contains extremely high levels of lipophilic components with low polarity and high viscosity and is nearly free of proteins, carbohydrates, and minerals. Although its matrix composition is uncomplicated, cereulide is a highly lipophilic toxin that readily dissolves in oil-phase substances. During organic solvent extraction, massive co-extraction of endogenous lipids inevitably occurs, causing severe emulsification, impurity co-elution, chromatographic column contamination, and intensive matrix effects, which substantially hinder accurate toxin quantification. In contrast, ARA powder is a microencapsulated product prepared by encapsulating lipid ARA oil with polymeric wall materials (e.g., maltodextrin, gum arabic, and whey protein) [13]. The water-soluble wall materials tend to swell, gelatinize, and agglomerate in aqueous environments, impeding organic solvent penetration and microcapsule rupture. Incomplete wall breaking further leads to residual encapsulated toxin and low extraction recovery.

Our research group has previously established a solid-phase extraction coupled with LC-MS/MS (SPE-LC-MS/MS) method for cereulide detection in ARA-fortified infant formula, accumulating valuable technical experience in matrix pretreatment and toxin quantitative analysis [20]. Infant formula represents a highly complex matrix containing various interfering substances, including milk proteins, lactose, vitamins, and minerals [21], which is fundamentally different from the relatively simple chemical compositions of ARA oil and ARA powder. In theory, the simpler matrix background of ARA oil and ARA powder can effectively reduce matrix interference during cereulide extraction, enrichment, and instrumental detection. However, the unique lipid characteristics of ARA oil and the special microencapsulated structure of ARA powder still adversely affect extraction efficiency and purification effectiveness. Furthermore, the EU mandates a 50% sampling rate for these raw materials, leading to massive sample throughput and a heavy analytical workload during routine surveillance. Therefore, targeted optimization of pretreatment procedures is essential to develop rapid, simple, and efficient extraction and purification methods that meet the regulatory demands for large-scale sample surveillance of cereulide in ARA materials.

Based on our prior research foundation in infant formula detection, the present study systematically optimized extraction and purification methods by integrating the specific matrix characteristics of ARA oil and ARA powder. By adopting optimized adsorbents combined with a pass-through purification cartridge, a reliable and efficient sample pretreatment method was successfully established for cereulide determination in two ARA matrices. This proposed method achieves rapid and high-efficiency purification of trace cereulide toxin, providing a reliable technical foundation for routine monitoring and risk control of cereulide contamination in ARA raw materials.

## 2. Results and Discussion

### 2.1. Optimization of Extraction Method

The selection of extraction solvent is a critical step in method development, directly influencing analyte extraction efficiency, matrix cleanup efficiency, accuracy, and sensitivity of subsequent detection. ISO 18465:2017 [22], the authoritative standard method published by the International Organization for Standardization, specifies ACN as the extraction solvent for cereulide. This parameter has been collaboratively validated by multiple international laboratories, exhibiting reliable method performance and favorable inter-laboratory data comparability. However, the optimal solvent-to-matrix ratio varies considerably across diverse matrices in existing studies [16,17,23,24], reflecting the need for matrix-specific optimization. Therefore, we adopted ACN as the extraction solvent and systematically optimized the solvent-to-matrix ratio to maximize the recovery of cereulide from ARA oil and ARA powder matrices.

**Figure 1:**
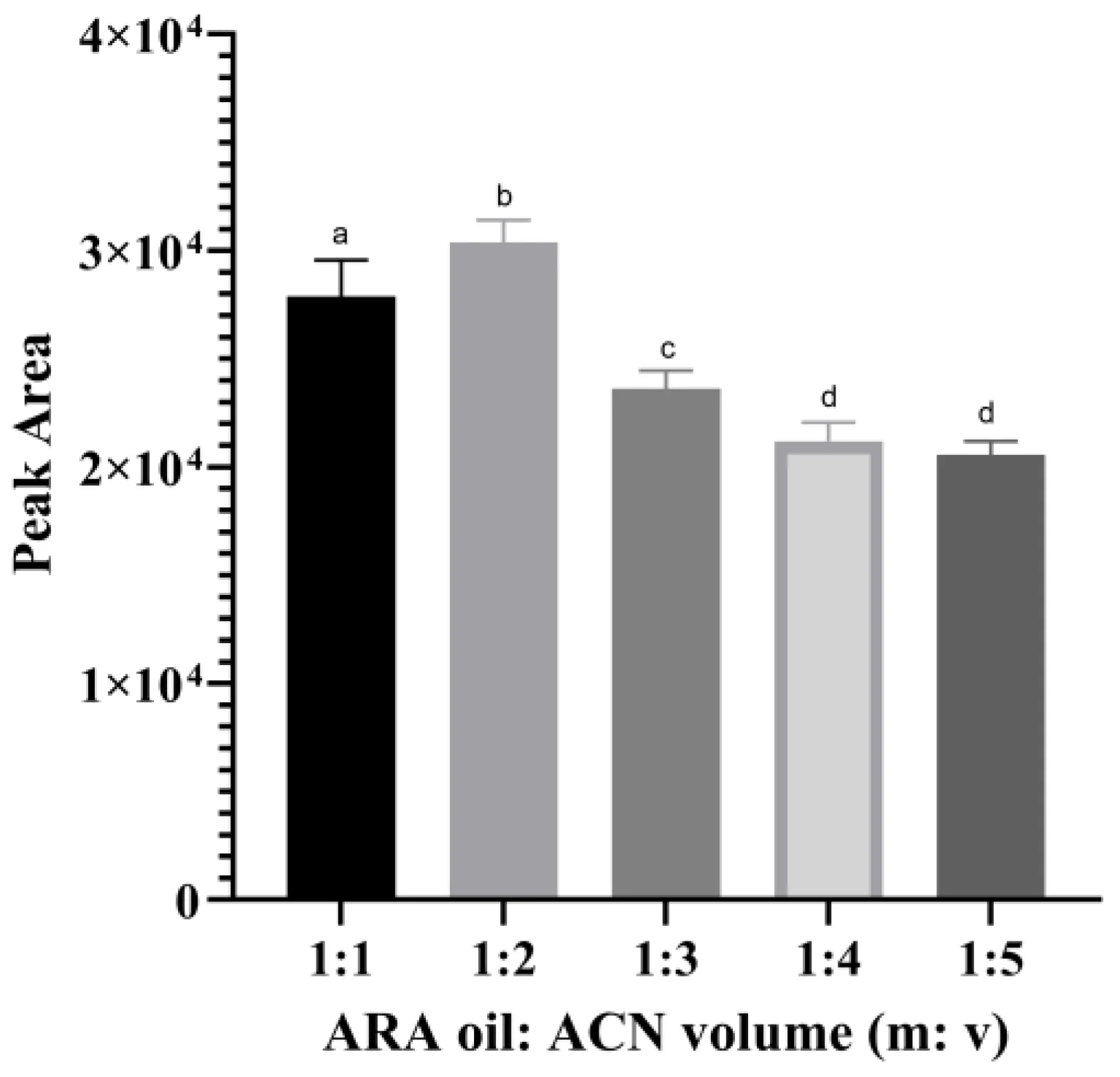
Influence of ARA oil-to-ACN ratio (m:v) on cereulide detection sensitivity. Results are presented as mean ± SD (*n* = 3). Different lowercase letters (a, b, c, d) indicate the results of multiple comparisons after one-way ANOVA; groups labeled with different lowercase letters are significantly different (*p* < 0.05).

For the ARA oil matrix, 1.00 g of homogenized sample was weighed, and the spiking level of cereulide was set at 1.0 μg·kg^−1^. We tested ACN extraction volumes of 1, 2, 3, 4, and 5 mL, yielding matrix-to-solvent ratios of: 1:1, 1:2, 1:3, 1:4, and 1:5 (ARA oil: ACN, m:v). The resulting extracts were purified, dried under nitrogen blowdown, and reconstituted in 1 mL of ACN for instrumental analysis. We evaluated extraction efficiency by comparing the chromatographic peak areas. The cereulide peak area varied with the ARA oil-to-ACN ratio (1:1 to 1:5, m:v), rising to a maximum at 1:2 and declining at 1:3, with no obvious variation from 1:3 to 1:5 (Figure 1). One-way ANOVA confirmed significant differences among all tested ratios (*p* < 0.05); the 1:2 ratio produced significantly higher recoveries than all other groups (*p* < 0.05), whereas the 1:3, 1:4, and 1:5 groups exhibited comparable extraction performance (*p* > 0.05). Therefore, a matrix-to-solvent ratio of 1:2 (m:v) was selected as the optimal condition to achieve the maximum extraction efficiency of cereulide.

ARA powder is a microencapsulated raw material, with its capsule wall materials composed primarily of water-soluble food-grade polymers such as maltodextrin, whey protein, and β-cyclodextrin. In an ACN extraction system, the absence of a hydration medium prevents these water-soluble wall materials from fully swelling and dispersing, leading to agglomeration and the formation of a dense encapsulation structure. This structure severely impedes contact between cereulide in the capsule core and the ACN extraction solvent, ultimately resulting in incomplete extraction and significantly underestimated quantitative results. Drawing upon protocols issued by the Austrian Agency for Health and Food Safety (AGES) and our previously established pretreatment technology for cereulide detection in ARA-fortified infant formula [20,24], we developed a stepwise extraction strategy: hydration dispersion followed by organic solvent precipitation extraction. Referring to the optimal conditions for infant formula samples, we fixed the sample-to-water ratio at 1:2 (m:v) for hydration and then optimized the subsequent ACN volume ratio. As this method employs a pure organic-phase pass-through purification step, NaCl was added before purification to induce a salting-out effect. This treatment enables rapid phase separation of organic and aqueous phases, reduces matrix interference, and ultimately guarantees the purification efficiency and detection accuracy of subsequent experiments.

**Figure 2:**
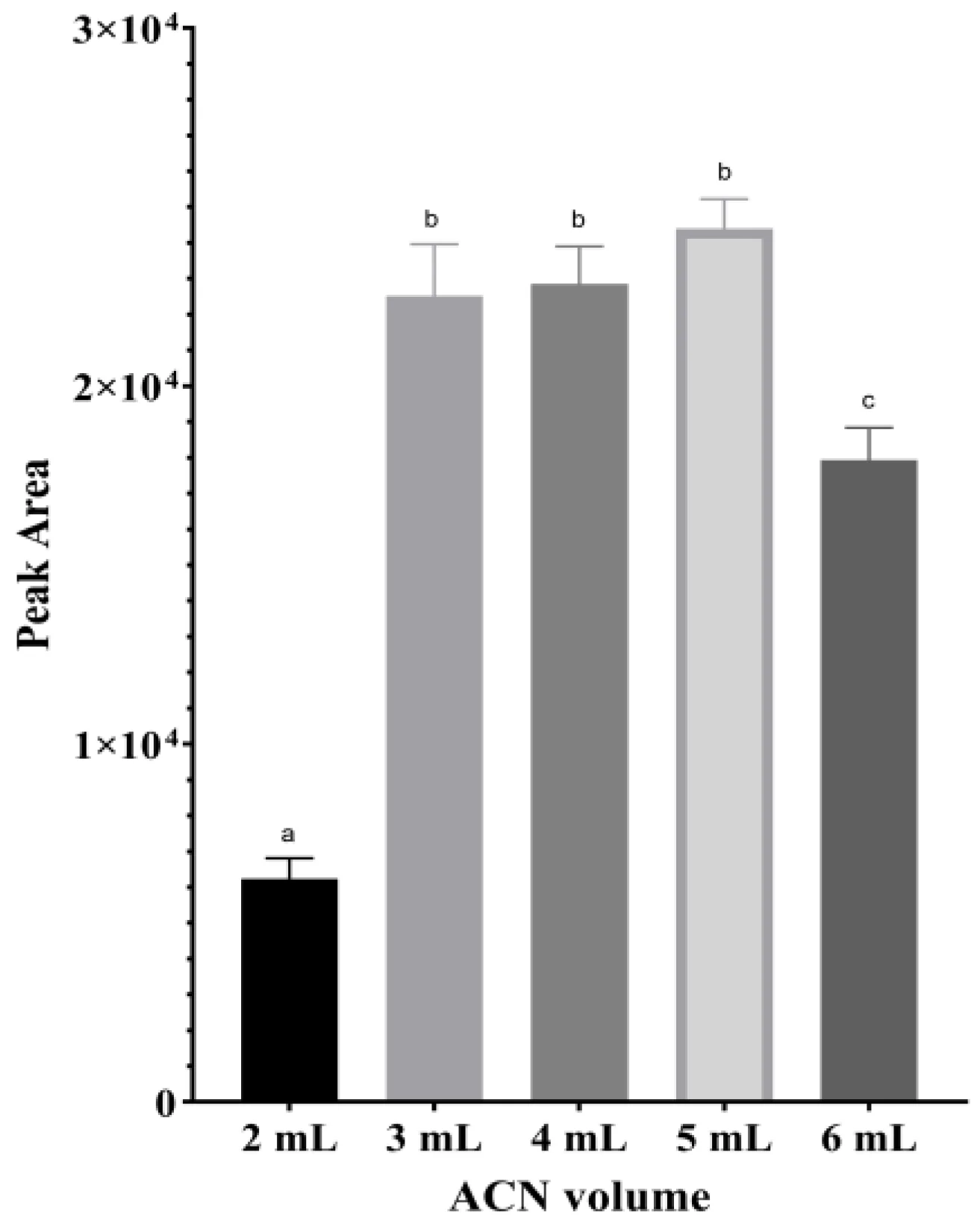
Influence of ACN volume on cereulide detection sensitivity in ARA powder. Results are presented as mean ± SD (*n* = 3). Different lowercase letters (a, b, c) indicate the results of multiple comparisons after one-way ANOVA; groups labeled with different lowercase letters are significantly different (*p* < 0.05).

For the ARA powder matrix, 1.00 g of homogenized sample was weighed and fortified with cereulide at a spiking level of 1.0 μg·kg^−1^. 2 mL deionized water was added to hydrate the matrix, and the mixture was thoroughly vortexed to achieve adequate swelling and dissociation of the microcapsule structure. Subsequently, different volumes of ACN (2, 3, 4, 5, and 6 mL) were added to denature proteins and extract cereulide. Afterwards, 0.5 g of NaCl was added to each sample to induce the salting-out effect and facilitate complete phase separation. Under the above experimental conditions, the resulting extracts were purified, dried under nitrogen blowdown, and reconstituted with 1 mL of ACN prior to instrumental detection. Accordingly, the variations in peak areas were compared to systematically assess the trends in extraction efficiency at the fixed spiking level of 1.0 μg·kg^−1^. As shown in Figure 2, increasing the ACN volume from 2 to 5 mL caused a marked increase in cereulide peak area. One-way ANOVA revealed that the ACN volume exerted a significant influence on cereulide extraction performance (*p* < 0.05). No statistically significant differences were observed among the 3, 4, and 5 mL groups (*p* > 0.05); the 5 mL group yielded the maximum peak area with a slight upward trend, indicating marginally improved extraction efficiency. Further raising the ACN volume to 6 mL markedly reduced the extraction efficiency (*p* < 0.05). Accordingly, 5 mL was selected as the optimal ACN volume to achieve the highest extraction efficiency.

Excess ACN volume leads to a reduction in the peak area of cereulide. This phenomenon can be comprehensively interpreted by combining the physicochemical properties of ACN and the compositional differences between ARA oil and ARA powder. As a solvent with intermediate polarity, ACN exhibits excellent extraction performance for cereulide. Nevertheless, satisfactory extraction efficiency can only be obtained by balancing extraction capability and selectivity to limit the co-extraction of matrix impurities.

Both matrices exhibited the same trends. Within the optimal ACN volume range, the cereulide signal intensity increased with increasing ACN volume, whereas further addition of excess ACN significantly attenuated the detection response. The ARA oil matrix consists solely of neutral lipids; excessive ACN co-extracts large amounts of lipids and triggers severe ion suppression. Similarly, excess ACN co-extracted additional neutral lipid impurities from hydrated ARA powder, and these co-extracted lipid contaminants induce pronounced matrix ion suppression. Although a larger solvent volume can theoretically enhance the total extraction of cereulide, aggravated matrix suppression overwhelmingly offsets the benefit, ultimately resulting in lower normalized extraction efficiency. Therefore, the reduction in extraction efficiency observed with excessive ACN volumes can be attributed to solvent saturation and the concomitant increase in co-extracted matrix interferences, leading to pronounced matrix effects. This finding is consistent with previous literature reports [25].

In summary, the optimal extraction conditions were determined as 1:2 (m:v, 1 g ARA oil with 2 mL ACN) for ARA oil, and a 1:2 (m:v) powder-to-water ratio for rehydration followed by extraction with 5 mL ACN and the addition of 0.5 g NaCl for salting-out for ARA powder. The optimized parameters established in this study achieve maximized detection sensitivity while ensuring satisfactory extraction recovery and minimal matrix effects.

### 2.2. Optimization of Clean-Up Procedure

Following the resolution of extraction challenges specific to the high-lipid ARA oil and microencapsulated ARA powder matrices, we further optimized sample purification conditions. Although ARA oil and ARA powder have relatively simple matrix compositions, the use of ACN, a moderate-polarity extraction solvent, inevitably co-extracts substantial lipophilic interferents together with cereulide. These co-extracted lipids caused pronounced matrix effects and a marked reduction in detection accuracy. Previous studies have demonstrated that zirconia-based materials exhibit excellent lipid scavenging capacity [26]. Moreover, our prior work confirmed that HLB-P, a copolymer sorbent modified with N-vinylpyrrolidone and divinylbenzene, achieves favorable purification efficiency for infant formula matrices containing ARA powder. On this basis, we comparatively evaluated two types of cleanup sorbents: HLB-P and inorganic zirconia-based HMR. Key purification parameters (operation mode and sorbent dosage) were systematically optimized to establish an optimal sample cleanup protocol.

**Figure 3:**
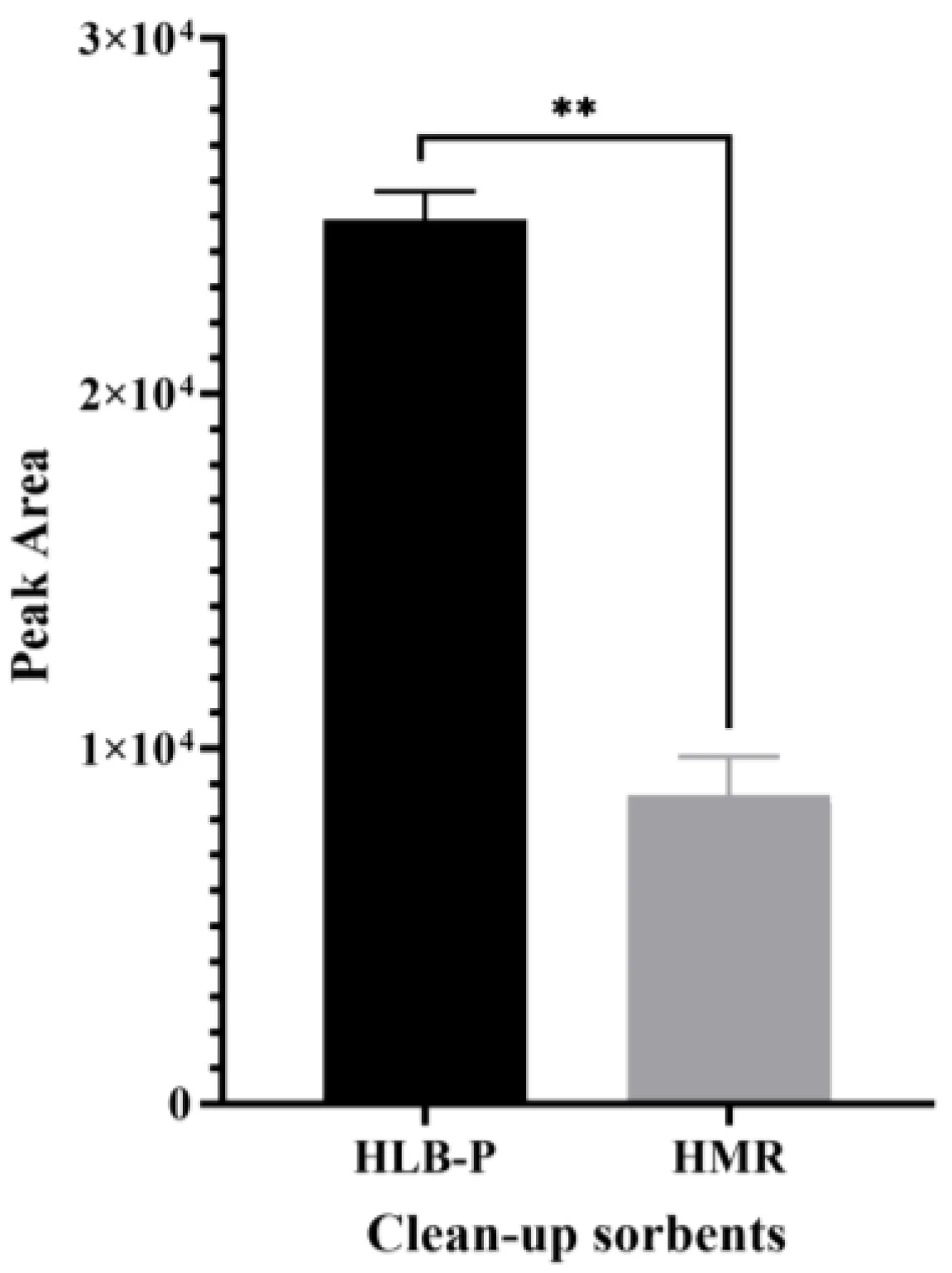
Comparison of the peak areas of cereulide after purification with HLB-P and HMR clean-up sorbents. Results are presented as mean ± SD (*n* = 3). ** indicates an extremely significant difference between the two groups (*p* < 0.01).

Under the optimized extraction conditions described above, the purification efficiencies of HLB-P and HMR sorbents were comparatively evaluated using ARA oil spiked with cereulide at 1.0 μg·kg^−1^. As shown in Figure 3, the peak area of cereulide after HLB-P purification was markedly higher than that acquired from samples treated with HMR sorbent (*p* < 0.01). The chromatographic peak area of cereulide purified by HLB-P reached approximately 2.5 × 10^4^, while the value for HMR was only around 8 × 10^3^. This marked difference in cereulide peak area indicates that HLB-P efficiently removed matrix interferents from ARA oil while preserving analyte recovery. In comparison, the HMR sorbent yielded a substantially lower cereulide peak response. This discrepancy can be attributed to the inferior retention capacity of HMR for lipophilic impurities inherent to the ARA matrix. After nitrogen blow-down, obvious lipid droplets were observed in HMR-purified extracts, which led to incomplete reconstitution of the extract and consequently impaired the quantitative detection of cereulide. For this reason, HLB-P was selected as the preferred sorbent for subsequent optimization of the cleanup procedure.

**Figure 4:**
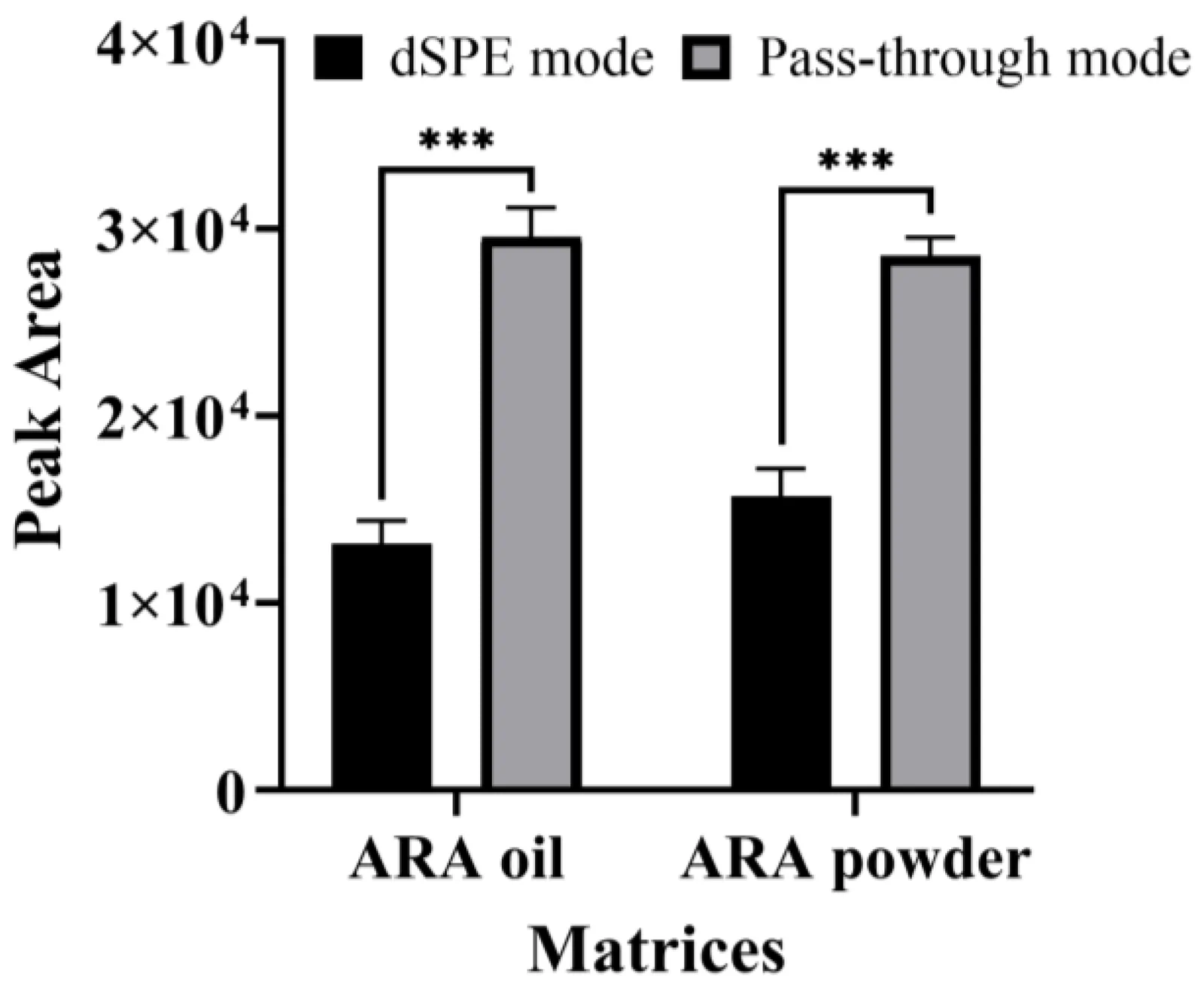
Comparison of cereulide detection sensitivity between dSPE and pass-through modes (200 mg HLB-P sorbent) for purification of ARA oil and ARA powder matrices. Results are presented as mean ± SD (*n* = 3). Asterisks indicate significant differences between the two purification modes: *** *p* < 0.001.

Given the relatively low matrix complexity of ARA oil and ARA powder, HLB-P was further assessed as the cleanup sorbent using two rapid pretreatment formats: pass-through cleanup and dispersive solid-phase extraction (dSPE). These approaches were evaluated with the aim of accelerating sample processing, minimizing experimental runtime, and reducing organic solvent consumption. In the dSPE protocol, HLB-P particles remained suspended in the extraction solvent and could not be completely sedimented even after high-speed centrifugation. As shown in Figure 4, under identical pretreatment conditions, the cereulide peak area obtained following dSPE purification was significantly lower than that acquired via HLB-P pass-through cleanup. One-way ANOVA confirmed that this difference was statistically significant (*p* < 0.01). These results indicated that the incomplete liquid-solid separation occurring in the dSPE mode resulted in considerable analyte loss. Consequently, the pass-through purification mode was ultimately adopted as the optimal cleanup strategy.

**Figure 5:**
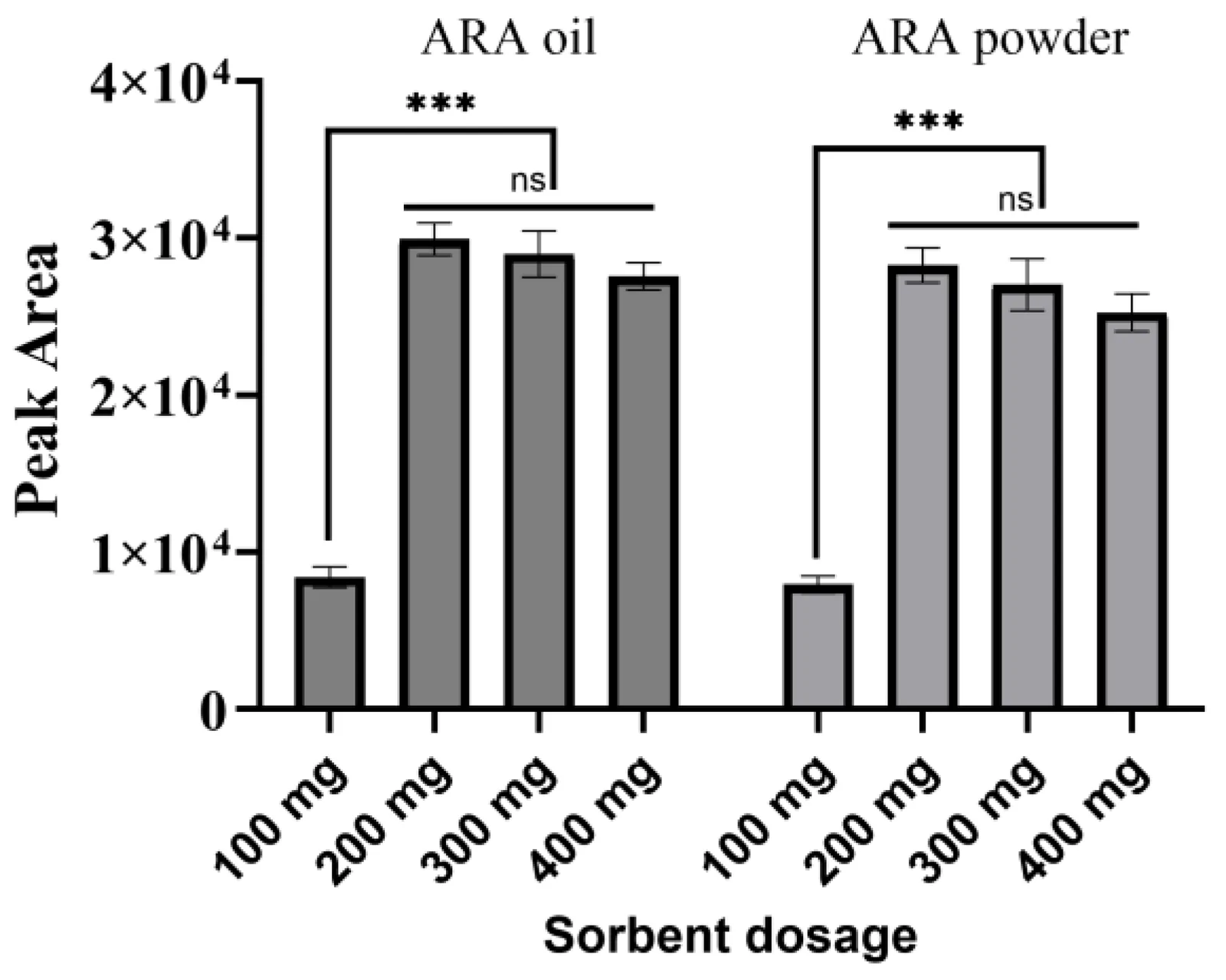
Influence of sorbent dosage on the sensitivity of cereulide detection in ARA oil and ARA powder. Results are expressed as mean ± SD (*n* = 3). “ns” indicates no significant difference; asterisks indicate significant differences between sorbent dosage: *** *p* < 0.001.

To further improve the cleanup performance, the loading dosage of HLB-P sorbent was systematically optimized in this section. Portions of 1.00 g ARA oil and ARA powder samples were fortified with cereulide at a spiking level of 1.0 μg·kg^−1^, respectively. All fortified samples were extracted according to the previously optimized extraction procedure, and the resulting crude extracts were further purified using HLB-P packed cartridges operated in pass-through mode to evaluate cleanup efficiency under different sorbent dosages. Four HLB-P sorbent loading levels (100, 200, 300, and 400 mg) were screened to determine the optimal sorbent dosage. As shown in Figure 5, the cereulide chromatographic peak area achieved with 200 mg HLB-P was significantly greater than that obtained using 100 mg sorbent. Further increasing the sorbent dosage from 200 to 400 mg induced no notable variation in cereulide peak response. One-way ANOVA verified that no statistically significant differences in peak area response were detected among the 200, 300, and 400 mg treatment groups (*p* > 0.05). Excess sorbent raises experimental consumption and detection cost without improving purification performance. Therefore, considering the principle of minimizing experimental cost while ensuring a satisfactory cleanup effect, 200 mg was determined as the optimal packed dosage of HLB-P sorbent.

### 2.3. Performance Validation

#### 2.3.1. Linearity

Linearity was assessed via calibration curves covering the concentration range of 0.1–20 μg·L^−1^. Seven calibration levels were set at 0.1, 0.5, 1.0, 2.0, 5.0, 10.0, and 20.0 μg·L^−1^, with each standard solution containing 0.5 μg·L^−1^ of the internal standard. Linear regression was performed by plotting the concentration ratio of cereulide to internal standard (*x*) against the corresponding peak area ratio (*y*). The method exhibited excellent linearity across the investigated range, yielding a correlation coefficient (R^2^) greater than 0.9998 with the regression equation *y* = 0.4805*x* + 0.0048. The EU sets a non-detectable regulatory threshold (<0.1 μg·kg^−1^) for cereulide in ARA materials. The lower limit of quantification (0.1 μg·kg^−1^) of the developed method complies with this regulatory standard, fully satisfying the sensitivity criteria for trace-level screening.

#### 2.3.2. Sensitivity

Under the optimized conditions, the limit of detection (LOD) and limit of quantification (LOQ) of cereulide were 0.03 μg·kg^−1^ and 0.1 μg·kg^−1^, respectively. This method exhibits favorable sensitivity for trace cereulide analysis in ARA oil and powder matrices. The LOD was further validated by analyzing 20 parallel spiked samples for each matrix. ARA oil and ARA powder were fortified at the LOD concentration of 0.03 μg·kg^−1^ (*n* = 20 per matrix). All samples yielded a 100% positive detection rate. This result confirms reliable detection at this critical concentration.

Notably, the LOQ of 0.1 μg·kg^−1^ complies with the EC limit for cereulide in ARA raw materials (<0.1 μg·kg^−1^) and allows accurate quantification at the compliance threshold. The LOD of 0.03 μg·kg^−1^ accounts for 30% of the regulatory limit and ensures adequate sensitivity for screening. The validated LOQ enables reliable confirmatory quantification. This method features a broad linear range of 0.1–20 μg·L^−1^. Its extensive linear working range fulfills routine regulatory compliance testing and the monitoring of highly contaminated ARA raw materials.

#### 2.3.3. Matrix Effect

**Figure 6:**
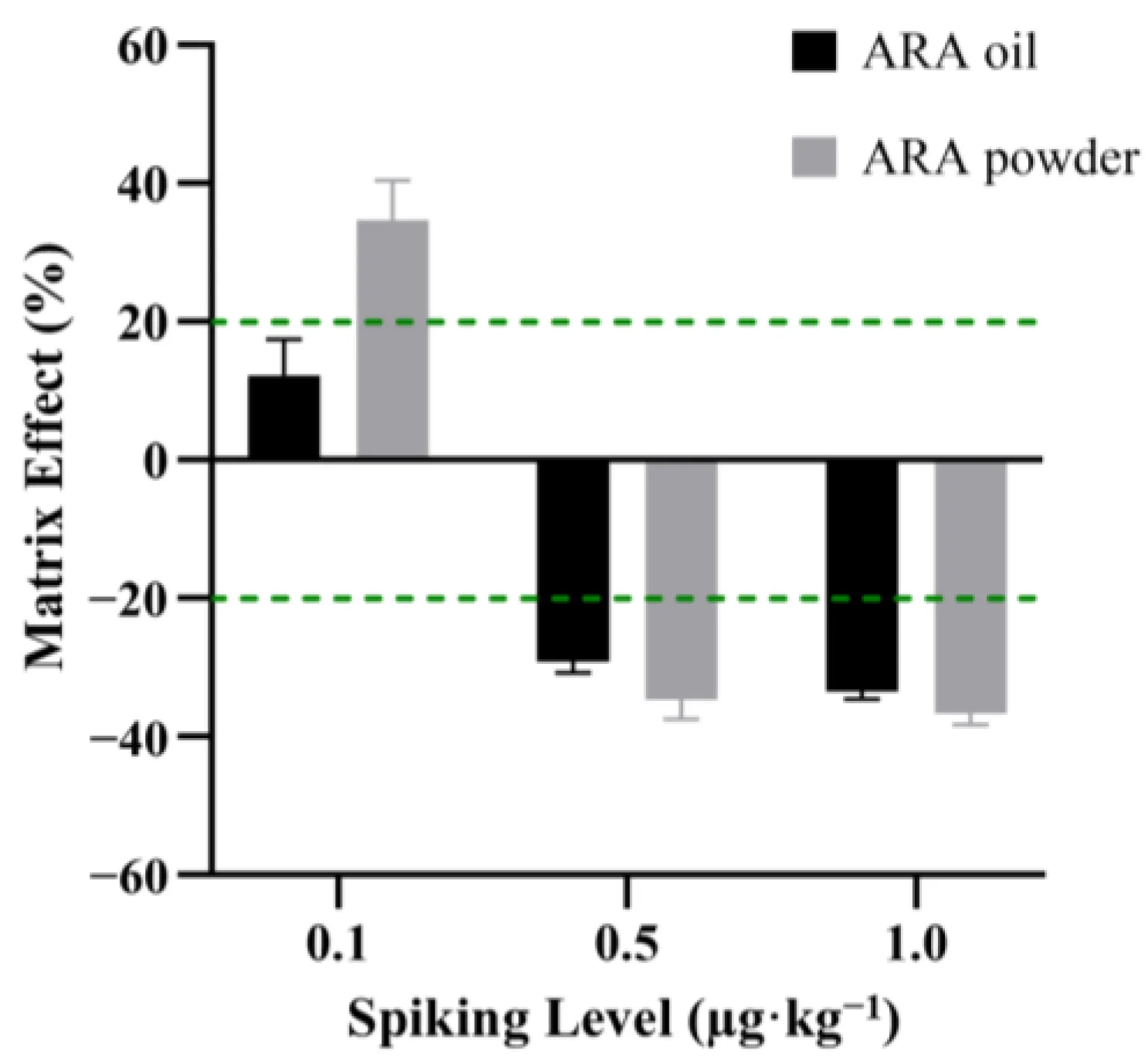
Matrix effects of cereulide in ARA oil and ARA powder at three levels. Results are expressed as mean ± SD (*n* = 3). Matrix effects were calculated by comparing the peak area ratios of matrix-matched standards with those of solvent-based standards at equivalent concentrations. The dashed lines indicate ± 20%, representing no matrix effect. Values > 20% indicate signal enhancement, and values < −20% indicate signal suppression.

In this study, matrix effects in ARA oil and ARA powder samples were systematically evaluated. As shown in Figure 6, both ARA oil and ARA powder exhibited concentration-dependent matrix effects. At the low concentration levels of 0.1 μg·kg^−1^, signal enhancement was observed for both ARA oil (12.2% ± 5.2%) and powder (34.7% ± 5.7%), indicating obvious ionization enhancement under diluted matrix conditions. ARA powder displayed a stronger enhancement effect compared with the oil matrix at this concentration level. In contrast, at 0.5 and 1.0 μg·kg^−1^, both matrices presented signal suppression. ARA oil showed matrix effects of −29.2% ± 1.6% and −33.5% ± 1.1%, while the ARA powder exhibited values of −34.7% ± 2.8% and −36.7% ± 1.6%. The magnitude of suppression remained relatively stable and comparable between the two ARA sample matrices at higher concentrations. It is well documented that matrix effects are critical determinants of quantitative accuracy in LC-MS/MS analysis, depending on both analyte properties and matrix composition [27]. Matrix effects are considered significant if they exceed ± 20% [28]. As shown in the results, both ARA oil and ARA powder exhibited moderate matrix effects. Nevertheless, matrix interferences were effectively compensated via internal standard calibration, as evidenced by the satisfactory spike recoveries (see Section 2.3.4), which ensured the quantitative accuracy of the established method.

#### 2.3.4. Trueness and Precision

Method trueness and precision were evaluated using spiked ARA oil and ARA powder samples at three concentration levels (0.1, 0.5 and 1.0 μg·kg^−1^), encompassing the LOQ, a mid-range concentration, and a higher concentration level. For ARA oil, spike recoveries ranged from 90.7% to 107% with relative standard deviations (RSDs) ≤ 6.5%; for ARA powder, recoveries ranged from 88.5% to 107.9% with RSD ≤ 7.0%. These results (shown in Table 1) demonstrate satisfactory method trueness, with mean recoveries falling within the acceptable range of 50–120% for trace analysis in complex food matrices, as recommended by the Codex Alimentarius Commission (CAC), EU Commission Decision 2002/657/EC guidelines, and GB/T 27404-2026 (Guidelines for Laboratory Quality Control in Physicochemical Analysis of Food in China). The intra-day precision, expressed as RSD, was consistently below 7.0% across all spiking levels and both matrices, indicating excellent repeatability under controlled analytical conditions.

## 3. Conclusions

In this study, a rapid UPLC-MS/MS method was established and validated for the determination of cereulide in ARA oil and ARA powder. Combined with optimized extraction parameters and a streamlined pass-through cleanup procedure using HLB-P sorbent, the improved sample pretreatment workflow substantially enhances the practicability of cereulide quantification in ARA raw material matrices. The proposed simplified and time-saving pretreatment reduces experimental runtime and organic solvent consumption while maintaining favorable cleanup performance. Comprehensive method validation confirmed that the established method satisfies international regulatory criteria and exhibits satisfactory sensitivity, trueness, and precision. Owing to its simplicity and rapidity, this approach elevates analytical efficiency and provides reliable technical support for the source safety supervision and risk control of infant food products.

## 4. Materials and Methods

### 4.1. Chemicals and Reagents

Acetonitrile, formic acid, and ammonium acetate for the preparation of mobile phases were of LC-MS grade and were purchased from ANAVO Scientific Co., Ltd. (Beijing, China). Sodium chloride (NaCl, GR grade) was obtained from Sinopharm Chemical Reagent Co., Ltd. (Shanghai, China). Deionized water used throughout the study was obtained from a Milli-Q system (Merck Millipore, Burlington, MA, USA).

HLB-P (a modified polymeric packing material based on a copolymer of N-vinylpyrrolidone and divinylbenzene) and HMR (a modified packing material based on inorganic zirconia) cartridges were purchased from ANAVO Scientific Co., Ltd. (Beijing, China). For UPLC-MS/MS analysis, chromatography separation was achieved on a Shim-pack Scepter C_18_ column (300 Å, 2.1 mm × 100 mm i.d., dp:1.9 µm) from Shimadzu (Shimadzu Corporation, Kyoto, Japan).

Standard solution of cereulide (C_57_H_96_N_6_O_18_, CAS No. 157232-64-9; 100 μg·mL^−1^ in ACN) and ^13^C_6_-cereulide internal standard solution (C_51_^13^C_6_H_96_N_6_O_18_, CAS No. 1487375-69-8; 20 μg·mL^−1^ in ACN) were purchased from Alta Scientific Co., Ltd. (Tianjin, China).

### 4.2. Preparation of Standard Solutions

Intermediate standard solutions of cereulide and ^13^C_6_-cereulide were prepared separately by diluting the respective original standard solutions with ACN to a concentration of 0.1 µg·mL^−1^. Working standard solutions were prepared by further diluting each intermediate solution with ACN to a final concentration of 0.01 µg·mL^−1^. All the standard solutions were stored at −20 °C and allowed to equilibrate to room temperature (~25 °C) prior to use.

### 4.3. Sample Preparation

#### 4.3.1. Sample Extraction

For ARA oil sample pretreatment, 1.00 g of homogenized ARA oil was accurately weighed and transferred into a 15 mL centrifuge tube. After the addition of 50 μL of internal standard working solution at a concentration of 0.01 μg·mL^−1^, the mixture was thoroughly homogenized via vortexing. The sample was subsequently extracted with 2 mL of ACN under continuous shaking for 30 min. Following extraction, the mixture was centrifuged at 12,000 rpm and 4 °C for 10 min, and the supernatant ACN layer was carefully collected into a clean tube for further purification treatment.

For the treatment of ARA powder samples, 1.00 g of homogenized ARA powder was accurately weighed into a 15 mL centrifuge tube and spiked with 50 μL of 0.01 μg·mL^−1^ internal standard working solution, followed by sufficient vortex mixing. The powder sample was first hydrated with 2 mL of deionized water and vortexed for 1 min. Subsequently, 5 mL of ACN and 0.5 g of NaCl were added to the mixture. The mixture was shaken continuously for 30 min to ensure adequate extraction, then centrifuged at 12,000 rpm and 4 °C for 10 min. The upper ACN supernatant was transferred into a clean tube for further purification.

#### 4.3.2. Purification Method

Supernatants derived from ARA oil and ARA powder matrices were purified individually via HLB-P pass-through extraction cartridges. After sample loading, the flow-through fraction was collected, and the cartridge was then eluted with 2 mL ACN. The flow-through fraction and the cartridge eluate were combined and evaporated to dryness under a stream of nitrogen in a 40 °C water bath. The dry residues were reconstituted in ACN to a fixed volume of 1 mL. After vortex mixing for 1 min and centrifugation at 12,000 r/min and 4 °C for 10 min, the supernatants were collected for instrumental analysis.

### 4.4. LC-MS/MS Analysis

Analyses were performed using the UPLC-MS/MS system, consisting of an ACQUITY UPLC system coupled to a Xevo TQ-S mass spectrometer (Waters Corporation, Milford, MA, USA). The conditions for liquid chromatographic separation and mass spectrometric detection were referenced to those optimized in our previous study [20]. The relevant parameters are detailed briefly as follows.

#### 4.4.1. Chromatographic Condition

Chromatographic separation was performed using a Shim-pack Scepter C18 column (300 Å, 2.1 mm × 100 mm i.d., dp:1.9 µm). The analysis was conducted at a constant column temperature of 40 °C with a flow rate set at 0.4 mL·min^−1^ and an injection volume of 2.0 μL. The binary mobile phase system comprised ACN as solvent A and an aqueous solution of 2 mmol·L^−1^ ammonium acetate containing 0.1% formic acid as solvent B. Gradient elution was implemented by linearly increasing the proportion of solvent A from 80% to 100% over the initial 4.0 min, holding the mobile phase composition at 100% solvent A from 4.0 min to 6.0 min, and then decreasing the proportion of solvent A back to 80% between 6.0 min and 7.0 min, after which the column was re-equilibrated up to 8.0 min prior to the subsequent sample injection.

#### 4.4.2. Spectrometry Condition

MS/MS detection was performed using an electrospray ionization (ESI) source. The optimized parameters were as follows: the capillary voltage was set to 3.2 kV, alongside a source temperature of 150 °C, desolvation temperature of 500 °C, desolvation gas flow rate of 1000 L/h, and a collision cell pressure of 0.36 Pa. Quantitative analysis was carried out under positive ion mode via multiple reaction monitoring (MRM), with relative MRM parameters adopted from the optimized conditions reported in our previous study [20]. All chromatographic and mass spectrometric data acquisition and processing were performed using MassLynx software (version V4.1, Waters Corporation, Milford, MA, USA). Statistical analysis was performed using GraphPad Prism version 11 and Microsoft Excel 2021.

### 4.5. Method Validation

The proposed method was fully validated by evaluating its key analytical performance characteristics, including linearity, limit of detection (LOD), limit of quantification (LOQ), matrix effect, trueness, and precision.

Linearity was assessed using calibration curves prepared in ACN within the concentration range of 0.1–20 µg·L^−1^. Seven calibration levels were set at 0.1, 0.5, 1, 2, 5, 10.0, and 20.0 µg·L^−1^_,_ with each standard solution containing 0.5 µg·L^−1^ of the internal standard. The correlation coefficient (R^2^) was used to evaluate the linear regression performance.

Matrix effect of cereulide in ARA oil and ARA powder was evaluated at three concentration levels (0.1, 0.5 and 1.0 µg·kg^−1^). This was achieved by comparing the peak areas of cereulide spiked into pre-extracted blank matrices with those of cereulide in pure solvent subjected to the entire sample preparation procedure. According to SANTE/2020/12830 rev.2, matrix effect was expressed as the percentage of signal suppression or enhancement, calculated using the following equation: Matrix effect (%) = [peak area (matrix)/peak area (solvent) − 1] × 100% [27]. Matrix effects are considered significant if they exceed ±20%. Each determination was performed in triplicate (*n* = 3), and results are presented as mean ± standard deviation (SD).

The LOD and LOQ were assessed from the results obtained for cereulide at the low spiking level, corresponding to signal-to-noise ratios (S/Ns) of 3 and 10, respectively. Method trueness and precision were validated by fortifying blank ARA oil and powder samples at three concentration levels (0.1, 0.5, and 1.0 µg·kg^−1^), followed by sample pretreatment according to the optimized analytical procedure. Trueness was expressed as recovery rate (%) by comparing the measured concentrations with spiked concentrations. Method precision was evaluated in terms of the relative standard deviation (RSD) of replicate measurements.

## Figures and Tables

**Table 1:** Trueness and precision data for cereulide in ARA oil and powder (*n* = 6).

Matrices	Spiking Level (μg·kg^−1^)	Recovery (%)	RSD (%)
ARA oil	0.1	98.5	6.5
	0.5	99.2	5.5
	1.0	97.8	3.8
ARA powder	0.1	97.5	7.0
	0.5	101.0	5.8
	1.0	97.0	4.2

## Data Availability

The original contributions presented in this study are included in the article. Further inquiries can be directed to the corresponding authors.
